# Reversible lesion in the splenium of the corpus callosum

**DOI:** 10.1002/brb3.1440

**Published:** 2019-10-06

**Authors:** Syuichi Tetsuka

**Affiliations:** ^1^ Department of Neurology International University of Health and Welfare Hospital Nasushiobara Japan

**Keywords:** apparent diffusion coefficient, cytokine, cytotoxic edema, reversible, splenium of the corpus callosum

## Abstract

**Aim of Review:**

The presence of isolated, reversible lesions in the splenium of the corpus callosum (SCC) is essential to confirm the diagnosis of mild encephalitis/encephalopathy. The lesions usually heal within a month after the onset of neurological symptoms. Magnetic resonance imaging (MRI) has increasingly been used as a diagnostic tool, which has led to the publication of an increasing number of case reports. These have highlighted some inconsistencies about encephalitis/encephalopathy. First, the condition is not always mild and may be severe. Second, reversible lesions in the SCC have been identified in various diseases and conditions other than viral encephalitis/encephalopathy. Third, lesions in SCC are not always completely reversible. On this note, this review describes the specific clinical and radiological features of encephalitis/encephalopathy.

**Findings:**

The reversible lesion in SCC is an MRI finding observable in a wide variety of diseases and conditions. Thus, it should be considered as a secondary change rather than a peculiar feature associated with mild encephalitis/encephalopathy. If reversible lesions are present in the SCC, the symptoms and prognosis are not necessarily favorable, with manifestations of encephalitis/encephalopathy varying from absent to severe. Neuroradiological features that appear as isolated high‐intensity signals on diffusion‐weighted images and a decreased apparent diffusion coefficient of the lesion might indicate a diagnosis of cytotoxic edema. Findings of previous studies suggest that cytokine‐mediated cytotoxic edema of the SCC may be an important pathophysiological manifestation of this condition.

**Conclusion:**

The reversible lesions in the SCC found on MRI are not exclusive to encephalitis/encephalopathy but may be secondary to other disorders.

## Graphical abstract

The reversible lesion in the SCC is an MRI finding in a wide variety of diseases and conditions. Findings of previous studies suggest that cytokine‐mediated cytotoxic edema of the SCC may be an important pathophysiological manifestation of this condition. The reversible lesions in the SCC found on MRI might be secondary to several disorders.

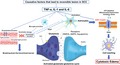

## 1 INTRODUCTION

Clinically mild encephalitis/encephalopathy with a reversible splenial lesion (MERS) is a clinical and radiological syndrome first proposed by Tada et al. in 2004. This phenomenon has come to be described more broadly as a clinical and radiological spectrum disorder called “reversible splenial lesion syndrome.” The clinical features of MERS are mild central nervous system disturbances such as seizure, confusion, and delirium. Affected patients usually recover completely without any sequelae within a month after the onset of neurological symptoms (Fang, Chen, Chen, Lin, & Yang, 2017; Tada et al., 2004; Takanashi, 2009). The radiological features of MERS include an appearance on magnetic resonance imaging (MRI) of an ovoid reversible lesion in the central portion of the splenium of the corpus callosum (SCC) in the acute phase. Sometimes, the lesion spreads throughout the whole corpus callosum and into the adjacent white matter without additional lesions, disappearing within approximately two weeks with neither residual signal changes on MRI nor atrophy (Hoshino et al., 2012; Tada et al., 2004; Takanashi et al., 2006). Because the use of MRI has become widespread, many cases of various conditions accompanied by MERS have been reported. One such condition is encephalitis/encephalopathy in which a reversible lesion is present in the SCC. Although MERS occurs in the context of mild encephalitis originally described in children, MERS as a terminology has been recently used in more adult reports and studies. As these case reports have increased in number, some inconsistencies about the associated disease concept have emerged. First, encephalitis/encephalopathy is not always mild but can be severe and isolated reversible lesions in the SCC are not always a good prognostic marker for a benign disease course in patients with MERS. However, in some cases, central nervous system disturbances are absent (Doherty, Jayadev, Watson, Konchada, & Hallam, 2005; Tetsuka & Ogawa, 2016; Zhang, Ma, & Feng, 2015). Second, a reversible lesion in the SCC has been recognized in patients with various diseases and conditions other than viral encephalitis/encephalopathy (Qing et al., 2019; Zhang et al., 2015). Third, the lesions are not always completely reversible (Doherty et al., 2005). In view of these three facts, a lesion in the SCC found on MRI might be secondary to other disorders. A similarity in neuroimaging features may suggest that there are common mechanisms that have different causes but lead to the same result. Thus, in this review, the clinical and radiological features that help clinically define the encephalitis/encephalopathy are described. The potential pathophysiological mechanisms of MERS and the associated pathological conditions are also discussed.

## 2 ANATOMY AND DEVELOPMENT

**Figure 1:**
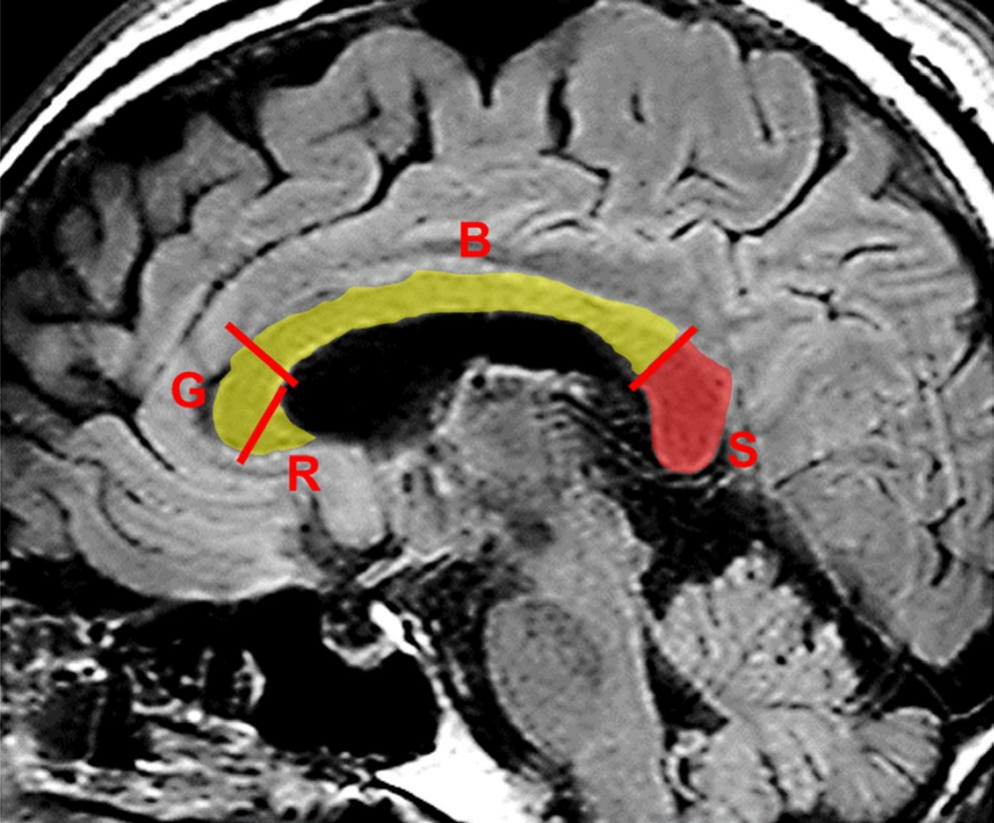
Midsagittal view of the splenium. Midsagittal fluid‐attenuated inversion recovery—weighted magnetic resonance image shows the corpus callosum and the splenium (S, in red). According to the conventional partitioning scheme, the splenium corresponds to the posterior 20% of the corpus callosum, which is separated by the border line perpendicular to the line linking the most anterior and posterior points of the corpus callosum. B, body; G, genu; R, rostrum

The corpus callosum is the major commissural area of the brain, consisting of white matter tracts of approximately 200 million myelinated nerve fibers that connect the left and right cerebral hemispheres. Anatomically from the anterior to the posterior, the corpus callosum comprises four parts: the rostrum, genu, body, and splenium, each connecting the bilateral cerebral hemispheres (Figure 1). The size of the fibers of the SCC that connect the posterior cortex varies: Those in the anterior part are thin, late‐myelinating fibers that mainly connect the parietal and temporal areas and those in the posterior part are thick, early‐myelinating fibers that link primary and secondary visual areas (Knyazeva, 2013). The function of the posterior fibers in the SCC is to connect the occipital lobes, enabling the processing of visual cues.

During brain development, at twelve to thirteen weeks of gestation, nerve fibers begin to cross the midline, giving rise to connections that later become the corpus callosum. During infancy, the corpus callosum expands rapidly as a result of an increase in the number of axons and myelin, and the development of the corpus callosum is complete by the age of four. Axonal myelination—the elaboration of myelin surrounding neuronal axons that is essential for normal brain function—begins at the age of 3–4 months in the SCC (Deoni et al., 2011). With regard to vascular distribution, the internal carotid artery network provides arterial blood supply to most of the corpus callosum, but the splenium receives arterial blood by the anterior pericallosal artery from the anterior circulation, and by the posterior pericallosal artery and posterior accessory pericallosal artery from the posterior circulation (Kahilogullari et al., 2013; Kakou, Velut, & Destrieux, 1998).

## 3 CAUSES OF LESIONS IN THE SPLENIUM OF THE CORPUS CALLOSUM AND INCIDENCE

Splenium of the corpus callosum lesions demonstrated on MRI in patients with viral encephalitis/encephalopathy were thought to be findings specific to MERS and MRI splenial lesions have been a good prognostic radiological marker for patients with encephalitis/encephalopathy (Hoshino et al., 2012; Takanashi, 2009). However, as increasing numbers of cases of MERS have been reported, the lesion has been found to be associated with various other diseases and conditions. Previously, this was often reported in children but it has become more recognized recently in adults that a reversible lesion in the SCC may no longer be a specific MRI finding in children with mild encephalitis. According to some case reports, MERS could have been triggered by (a) viral infection, including those caused by the influenza virus (Takanashi, Barkovich, Yamaguchi, & Kohno, 2004; Takatsu, Ishimaru, Ito, & Kinami, 2017), rotavirus (Fuchigami et al., 2013), measles virus (Melenotte, Craighero, Girard, Brouqui, & Botelho‐Nevers, 2013), adenovirus (Hibino et al., 2014), human parvovirus B19 (Suzuki, Kusaka, & Okada, 2014), and cytomegalovirus (Feraco, Porretti, Marchiò, Bellizzi, & Recla, 2018) and (b) other types of infectious pathogens including *Mycoplasma pneumoniae* (Yuan et al., 2016), *Legionella pneumophila* (Tomizawa et al., 2015), *Streptococcus pneumoniae* (Avcu, Kilinc, Eraslan, Karapinar, & Vardar, 2017), and malaria parasites (Mawatari, Kobayashi, & Yamamoto, 2018).

In addition to infection, MERS has also been reported to be associated with epilepsy and the use of antiepileptic drugs (Maeda et al., 2003; Mirsattari, Lee, Jones, & Blume, 2003). Extant literature has reported the other potential causes including substance withdrawal, metabolic disturbance, drug‐related toxicity, malignancies, cerebrovascular diseases, traumatic brain injury, status migrainosus, and high‐altitude disease (altitude sickness) (Al Brashdi & Albayram, 2015; Bin & Lee, 2011; Garcia‐Monco et al., 2011; Kallenberg et al., 2007; Kim & Gean, 2011; Maeda et al., 2006; Park et al., 2017; Renard, Bonafe, & Heroum, 2007; Samanta, 2015; Starkey, Kobayashi, Numaguchi, & Moritani, 2017; Takanashi et al., 2009; Takayama, Kobayashi, Sugishita, & Mihara, 2000; Tha et al., 2002; Yamaguchi et al., 2019). A listing is provided in Table 1. Regarding the frequency of etiologies, Garcia‐Monco et al. reported the etiology for 113 cases with lesions in SCC. The common associated conditions in their order of magnitude were epilepsy and related‐disease (43.36%), infection (33.63%), other uncommon reasons (10.62%), metabolism‐related conditions (5.31%), and high‐altitude edema (7.80%) (Garcia‐Monco et al., 2011). A recent retrospective study identified the etiologies for 30 cases including cerebral infarction (50%), trauma (13.3%), tumor (10%), alcohol abuse (6.7%), seizure (6.7%), heat stroke (3.3%), multiple sclerosis (3.3%), drug intoxication (3.3%), and panhypopituitarism (3.3%). In all of these, cerebral infarction was the most common etiology (Park, Hwang, Jung, Hong, & Kwon, 2014).

**Table 1:** Causative factors that lead to reversible lesion in the splenium of the corpus callosum

Infection
Viral: influenza, rotavirus, measles, adenovirus, human parvovirus B19, cytomegalovirus, varicella‐zoster, adenovirus, rubella, human herpesvirus‐6, human herpesvirus‐7, human immunodeficiency virus, mumps, parainfluenza, enterovirus, Epstein–Barr virus
Bacterial: *Legionella pneumophila*, *Streptococcus pneumoniae*, *Salmonella enteritidis*, *Escherichia coli*, *Enterococcus faecalis*, *Klebsiella pneumoniae* (febrile urinary tract infection), *Campylobacter jejuni*
Other types of infection: *Mycoplasma pneumoniae*, malaria, dengue fever
Antiepileptic drug related
Antiepileptic drugs (e.g., carbamazepine, phenytoin, valproate, and lamotrigine)
Withdrawal (antiepileptic drugs)
Other pharmacological agents and toxic substances
Methyl bromide exposure, anticancer agent (5‐fluorouracil, cisplatin, and carboplatin), corticosteroids, metronidazole, tetracycline, intravenous immunoglobulin, alcoholism, carbon monoxide poisoning
Metabolic disturbance and associated disease
Hypoglycemia, hypernatremia, hyponatremia, Marchiafava–Bignami disease, hemolytic–uremic syndrome, thyroid storm, Wernicke encephalopathy, vitamin B12 deficiency
Functional brain diseases
Epilepsy (partial, secondarily, and generalized), status migrainosus, high‐altitude disease (altitude sickness), transient global amnesia
Malignancies
Lymphocytic leukemia, glioblastoma, spinal meningeal melanocytoma
Cerebrovascular diseases or vasculitis
Subarachnoid hemorrhage, ischemic stroke, Kawasaki disease
Traumatic brain injury; diffuse axonal injury
Autoimmune encephalitis, *N*‐methyl‐d‐aspartate receptor encephalitis, autoimmune thyroid disease, antivoltage‐gated potassium channel autoantibody syndrome, systemic lupus erythematosus
Miscellaneous Conditions
Mumps vaccine, radiation therapy, renal failure, preeclampsia, anorexia nervosa, malnutrition, sympathomimetic‐induced kaleidoscopic visual illusion syndrome, Charcot–Marie–Tooth disease

The reversible lesion in the SCC is caused by various and numerous diseases and conditions. According to a study of brain MRI scans of 450 patients with various medical conditions, the estimated prevalence of lesions in the SCC was approximately 3% (McLeod, Williams, Machen, & Lum, 1987). Park et al. retrospectively reviewed 5,078 consecutive brain MRIs showing abnormal lesions. In a 42‐month study period, 30 consecutive patients with SCC lesions on their brain MRI were identified (Park et al., 2014). With regard to epilepsy, it has been reported that of preoperatively evaluated patients with drug‐resistant epilepsy in whom rapid reduction of their antiepileptic drugs led to seizures or ictal electroencephalographic discharges, 0.7% had a reversible lesion in the SCC (Nelles, Bien, Kurthen, Falkenhausen, & Urbach, 2006). In addition, splenial lesions found on MRI were reported in 6 of 1,200 patients with epilepsy (Kim et al., 1999). These lesions account for approximately 16% of all cases of acute encephalopathy and are reported to be second only to acute encephalopathy as the cause of biphasic seizures and late reduced diffusion in Japan.

It is difficult to ascertain the precise frequency of this syndrome. However, it may be underestimated because MRI examination is not being performed in all patients with many of the different etiologic conditions. Greater MRI availability and an increasing awareness of reversible lesions in the SCC might cause more cases to be diagnosed incidentally. Thus, it may be better to think of the reversible lesion as secondary to those causes rather than a specific MRI finding of mild viral encephalitis. However, the splenium is specifically involved in many cases of encephalitis/encephalopathy, particularly with influenza virus infection, in comparison with other causes.

## 4 CLINICAL SPECTRUM AND MANIFESTATIONS

The clinical symptoms of the conditions accompanying the reversible lesion in the SCC are nonspecific and diverse, mainly because they reflect a wide spectrum of conditions that are suggestive of encephalopathy or encephalitis. A fever that precedes or accompanies neurological symptoms is the most common prodromal manifestation. Other general clinical symptoms include headache and digestive tract disturbances (vomiting and diarrhea) (Chen et al., 2016; Takanashi, 2009). Cognitive impairment, seizures, behavior changes, drowsiness, confusion, acute urinary retention, and delirium are common neurological symptoms associated with reversible lesions in the SCC, as reported in previous clinical studies (Chen et al., 2016; Tada et al., 2004; Yuan et al., 2017; Zhang et al., 2015). Other neurological symptoms include motor deterioration, slurred speech, neck stiffness, coma, tremor, ataxia, somnolence, dysarthria, visual disturbance, and dizziness (Chen et al., 2016; Tada et al., 2004; Yuan et al., 2017; Zhang et al., 2015).

In the disease concept of MERS, neurological symptoms are expected to disappear completely within a month and isolated reversible lesions in the SCC are considered to be a good prognostic marker for a benign disease course in affected patients (Tada et al., 2004; Takanashi, 2009). However, reversible lesions in the SCC do not necessarily indicate a favorable prognosis in cases of encephalitis/encephalopathy. Indeed, the prognosis for patients with a severe disturbance of consciousness is unfavorable, including death as a probable outcome (Doherty et al., 2005; Zhang et al., 2015). In contrast, there have been case reports of patients who presented with only fever or headache and without neurological symptoms (Doherty et al., 2005; Tetsuka & Ogawa, 2016; Tsuji, Yoshida, Miyakoshi, & Haruta, 2009). This variation in clinical symptoms raises the question of whether reversible lesions in the SCC do indeed represent a specific marker for mild encephalitis/encephalopathy. In addition, these results may suggest that physicians should not diagnose or initiate treatment for encephalitis/encephalopathy based merely on a lesion in the SCC revealed by MRI.

Direct brain damage in the SCC, for example, that results from splenium infection, is also manifested by variable neurological signs and symptoms such as confusion, ataxia, dysarthria, seizure, hemiparesis, sensory abnormality, and aphasia. Most patients with SCC infarctions, however, show nonspecific symptoms that, in some cases, are restricted to vertigo or headache (Doherty et al., 2005; Li et al., 2015). However, these neurological signs and symptoms are almost consistent with those caused by MERS; thus, the clinical manifestations of MERS, though nonspecific, may represent a transient dysfunction of the SCC.

## 5 NEURORADIOLOGICAL FEATURES

**Figure 2:**
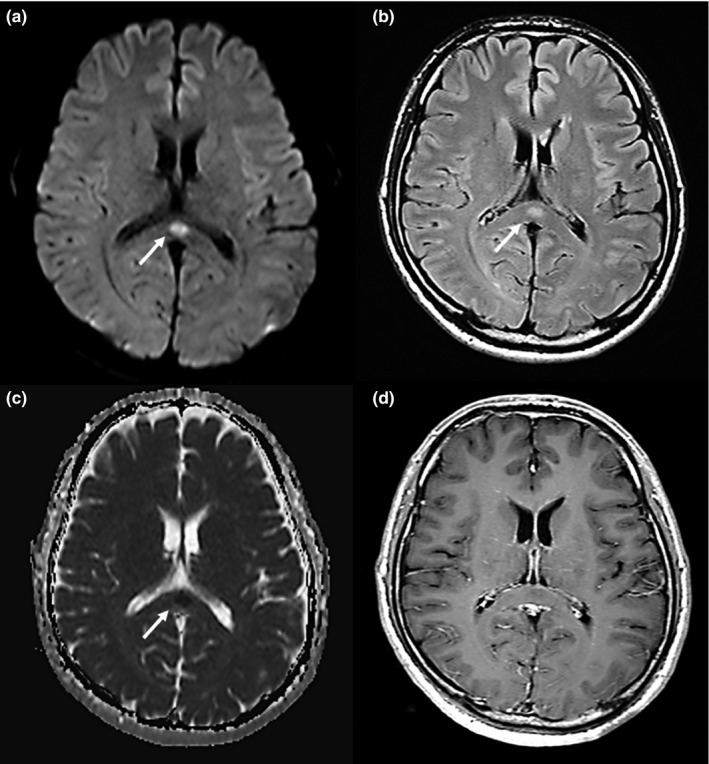
A 41‐year‐old man was admitted with fever, headache, and fatigue. He received a diagnosis of clinically mild encephalitis. Magnetic resonance imaging was performed on day 1 hospitalization. Diffusion‐weighted (a) and fluid‐attenuated inversion recovery (b) imaging showed a marked hyperintense signal (arrows) on the splenium of the corpus callosum (SCC). The apparent diffusion coefficient (c) showed a hypointense signal (arrow). T1‐weighted imaging with contrast material (d) showed an isointense signal that was not enhanced by the contrast material. Arrows indicate the SCC

**Figure 3:**
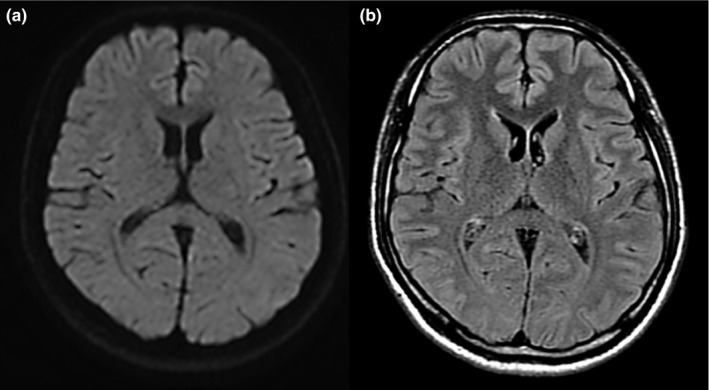
Magnetic resonance images on day 10 of hospitalization of the patient described in Figure 2. Axial diffusion‐weighted image (a) and fluid‐attenuated inversion recovery image (b) showed either disappearance or remarkable shrinking of the previously observed abnormalities. The patient was discharged without sequelae or medication

Typical MRI features are reversible hyperintense signals on T2‐weighted images, fluid‐attenuated inversion recovery images (FLAIR), and diffusion‐weighted images (DWI). In addition, the apparent diffusion coefficient (ADC) of the lesion is decreased on ADC maps, and hyperisointense signals may appear on T1‐weighted imaging sequences without contrast enhancement (Figure 2; Tada et al., 2004; Takanashi, 2009; Takanashi et al., 2006). The most characteristic MRI finding is reversible restricted diffusion, which after onset indicate the complete disappearance of the lesion or a clear reduction without atrophy in the lesion size and signal intensity within approximately 2 weeks (Figure 3; Hoshino et al., 2012; Tada et al., 2004; Takanashi et al., 2006; Zhang et al., 2015). In addition, the lesion of the corpus callosum is typically classified as having one of two patterns: (a) a small round or oval lesion, isolated in the center of the SCC in some patients with encephalitis/encephalopathy (clinically regarded as type 1 MERS) and (b) a lesion in the SCC expanding to the callosal fibers and into the adjacent cerebral white matter or a lesion in the SCC extending into the anterior portion of the corpus callosum (type 2 MERS) (Starkey et al., 2017; Takanashi et al., 2006; Takanashi, Imamura, Hayakawa, & Terada, 2010). Yang et al. used a quantifying ADC values method to study the extended range of type I MERS lesions. Their results showed that in the acute stage of MERS, not only the SCC but also the genu of the corpus callosum (GCC) region showed mild diffusion restriction. GCC diffusion restriction showed a recovery as it does in the SCC after a period. This suggests that the lesions in type I MERS may be more widespread than previously thought (Qing et al., 2019).

These MRI findings, however, are not specific to encephalitis/encephalopathy as they are seen in other diseases and conditions (Table 1). Both vasogenic and cytotoxic edema show signal hyperintensity on T2‐weighted images and on FLAIR sequences. However, DWI shows only markedly high density for cytotoxic edema and can show image contrast, which is dependent on the molecular movement of water. DWI is most useful for detecting conditions that are related to relative restriction of water diffusion such as increased cellularity, ischemia, viscous or mucinous degeneration, and intramyelinic sheath edema. It is also often used to diagnose acute cerebral infarction that results in cytotoxic edema that is considered to lead to an irreversible lesion and cellular death (Baehring & Fulbright, 2012). ADC maps display only the diffusion component; the SCC appears hypointense, and the low‐signal area in ADC generally indicates cytotoxic edema secondary to ischemia, which could lead to neurological sequelae (Starkey et al., 2017). However, in these disease conditions, the reversible lesions in the SCC usually disappear completely without sequelae. In contrast, hyperintensity on ADC maps generally indicates vasogenic edema that causes central nervous system vasculitis (Table 2; White, Hadley, Zhang, & Dogar, 2007).

**Table 2:** Characteristics of the reversible lesion in splenium of the corpus callosum

Image conditions (MRI)	Vasogenic edema	Cytotoxic edema	Reversible lesion in the SCC	Cerebral infarction (acute stage)
FLAIR	**↑**	**↑**	**↑**	N, then **↑**
DWI	N	**↑**	**↑**	N, then **↑**
ADC	**↑**	**↓**	**↓**	**↓**

Abbreviations: ↑, hyperintense signal; ↓, hypointense signal; ADC, apparent diffusion coefficient; FLAIR, fluid‐attenuated inversion recovery imaging; MRI, magnetic resonance imaging; N, normal; SCC, splenium of the corpus callosum.

## 6 PATHOPHYSIOLOGICAL HYPOTHESIS

The exact pathophysiological mechanism underlying the reversible lesion in the SCC is still obscure. There are several hypotheses about entities such as intramyelinic edema (Tada et al., 2004), inflammatory infiltrates (Tada et al., 2004), hyponatemia (Takanashi et al., 2009), oxidative stress (Miyata et al., 2012), neuroaxonal damage (Motobayashi et al., 2017), autoimmune processes (Kaminski & Prüss, 2019), and cytotoxic edema (Garcia‐Monco et al., 2011; Starkey et al., 2017). Cytotoxic edema is characterized by the swelling of the cellular elements in neurons and glial cells with a subsequent reduction in the extracellular space in the gray matter. Cytotoxic edema can also occur in glial cells, axons, and myelin sheaths of the white matter, the edema being localized in either the myelin sheath itself or the intramyelinic cleft. Thus, intramyelinic edema is a form of cytotoxic edema that is seen in the acute phase of multiple sclerosis, toxic or metabolic leukoencephalopathy, and osmotic myelinolysis. It has been proposed that intramyelinic edema may occur transiently in the SCC, where myelinated nerve fibers are dense rather than cytotoxic edema because intramyelinic edema causes a reduction in the ADC, does not lead to neuronal damage and is usually reversible. DWI abnormalities, however, resolve after the causative pathological factors are removed (Tada et al., 2004). In neonates with mild encephalopathy/encephalitis who do not have myelination in the SCC as mentioned previously, reversible lesions occur. As such, intramyelinic edema cannot provide a unified explanation (Sun et al., 2017). On the other hand, most cases of reversible lesions in the SCC demonstrate the evidence that cytotoxic edema of the SCC is an important mechanism in the condition (Garcia‐Monco et al., 2011; Starkey et al., 2017). Cytotoxic edema in the neurons, astrocytes, and oligodendrocytes of the SCC, caused by cytokines, may offer the best explanation. Recently, these lesions have been called “cytotoxic lesions of the corpus callosum” or “CLOCC” lesions (Galnares‐Olalde et al., 2019; Starkey et al., 2017).

The pathophysiological hypothesis about the reversible lesion in the SCC that leads to cytotoxic edema through cytokines is as follows: At first, cytokines such as tumor necrosis factor alpha, interleukin‐1, and interleukin‐6 are released under causative conditions that lead to reversible lesions in the SCC. These cytokines induce the expression of adhesion molecules (intercellular adhesion molecule 1 and vascular cell adhesion molecule) that interact with leukocytes. Next, the adhesion molecules make leukocytes produce reactive oxygen species and proteases that cause endothelial damage. Endothelial damage causes the activation of microglia, which accelerates the further release of these cytokines (Xing, Li, Deng, Ning, & Lo, 2018). Tumor necrosis factor alpha and interleukin‐1 can also induce astrocytes to produce vascular endothelial growth factor, which weakens the tight junction of the brain vasculature and results in the breaking down of the blood–brain barrier. In addition, interleukin‐1 can induce astrocytes to take up glutamate, which reacts with ammonia to form glutamine as influenced by the activity of glutamine synthetase. After that, glutamine is transported to the extracellular fluid where it is taken up by neurons and the glutamate synthesis process resumes. This sequence is referred to as the glutamate–glutamine cycle and it increases levels of extracellular glutamine (Zhou & Danbolt, 2014).

**Figure 4:**
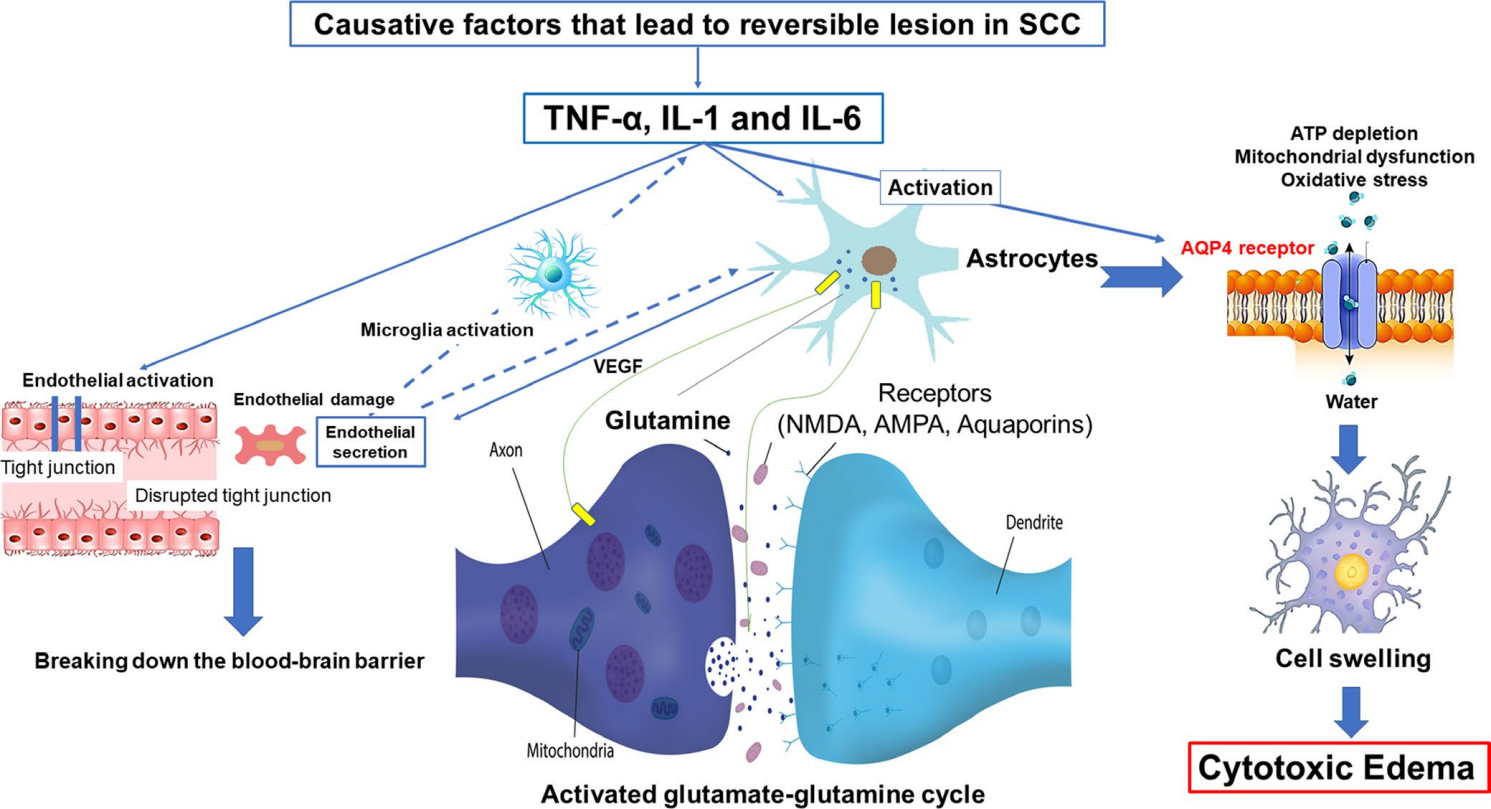
Depiction of the pathophysiological hypothesis of the reversible lesion in the splenium of the corpus callosum (SCC) that leads to cytotoxic edema. Cytokines, such as tumor necrosis factor alpha (TNF‐α), interleukin‐1 (IL‐1), and interleukin‐6 (IL‐6), are released. Endothelial damage causes activation of microglia that further accelerates the release of these cytokines. IL‐1 can also induce astrocytes to take up glutamate, which reacts with ammonia to form glutamine, and then glutamine is transported to the extracellular fluid, where it is again taken up by neurons; then the glutamate synthesis process restarts, thus increasing extracellular glutamine. Intracellular ATP depletion, resulting in mitochondrial dysfunction and oxidative stress, is induced by activated glutamate–glutamine cycle. The excitotoxic action of receptors, such as *N*‐methyl‐d‐aspartate (NMDA), α‐amino‐3‐ hydroxy‐5‐methyl‐4‐isoxazole propionic acid (AMPA), and aquaporin 4 (AQP4) receptors activation, is triggered through a complex cell–cytokine, that results in an influx of water into both astrocytes and neurons, which leads to cytotoxic edema, characterized by intracellular accumulation of fluid and Na^+^ and resulting in cell swelling. VEGF, vascular endothelial growth factor

Intracellular ATP depletion that leads to mitochondrial dysfunction and oxidative stress is induced by activated the glutamate–glutamine cycle (Haldipur et al., 2014; Rodrigo & Felipo, 2007). The excitotoxic action of receptors such as metabotropic glutamate receptors, glutamate on *N*‐methyl‐d‐aspartate, α‐amino‐3‐ hydroxy‐5‐methyl‐4‐isoxazole propionic acid receptors, and aquaporins results in an influx of water into both astrocytes and neurons. These events cause a disturbance of the intra‐extracellular ion balance. As a result, an excessive inflow of extracellular fluid and Na+ into cells is induced, leading to cellular swelling. These mechanisms ultimately result in cytotoxic edema (Figure 4; Liang, Bhatta, Gerzanich, & Simard, 2007).

Cytotoxic edema is characterized by the intracellular accumulation of fluid and Na^+^, which results in cell swelling. However, cytotoxic edema is thought to lead to an irreversible lesion and cellular death. While this is contrary to the most notable characteristics of reversible lesions in the SCC, the lesions are not always completely reversible (Doherty et al., 2005; McLeod et al., 1987). In the case of ischemia, neurons in the penumbra undergo cytotoxic edema that are potentially reversible if perfusion is restored within the first few hours but with cytotoxic edema caused by cytokine release, the margin of time to recovery is longer than that for ischemia. In addition, the lesion in SCC tends to show signs of recovery on MRI findings but there may be inflammatory tissue damage histopathologically (Starkey et al., 2017).

The SCC may have more abundant blood flow and be susceptible to cytotoxic edema caused by cytokine release in the blood than are other brain areas because the SCC is supplied not only by the anterior circulation but also from the posterior circulation, unlike the rest of the corpus callosum that is solely supplied by the internal carotid artery network (Kahilogullari et al., 2013; Kakou et al., 1998). In the neurons, astrocytes, and oligodendrocytes of the corpus callosum, there is also a higher density of receptors including cytokine receptors and glutamate (Hassel, Boldingh, Narvesen, Iversen, & Skrede, 2003). This higher density may be more likely to cause cytotoxic edema in the corpus callosum when cytokines release occurs. This has been experimentally proven that as follows: N‐methyl‐D‐aspartate receptors are expressed in the CC and are involved in excitotoxic activity in CC slices in vitro (Zhang, Liu, Fox, & Xiong, 2013). Moreover, there is also a higher density of aquaporin 4 (AQP4) receptors in the SCC that can lead to the development of cytotoxic edema (Badaut, Fukuda, Jullienne, & Petry, 2014). Recently, Yu et al. propose pathogenic mechanism for the reversible lesion in the SCC as related to AQP4 receptors as follows (Qing et al., 2019; Yang, Chang, Liu, & Yu, 2019): Various of conditions which have been reported to trigger the reversible lesion in the SCC (Table 1) can also affect the AQP4 protein expression, leading to an increased expression levels through a complex cell–cytokine interaction. As the basis for that mechanism, there are previous animal experiments that cytokine (interleukin‐1β) induces the activation of AQP4 through a the nuclear factor‐κB pathway in the rat brain (Ito et al., 2006), accelerated progression of cytotoxic brain edema was recognized in transgenic mice (AQP4‐ overexpressing) (Yang, Zador, & Verkman, 2008), and the absence of AQP4 was associated with decreased mortality and increased motor recovery after transient cerebral ischemia in AQP4‐knockout mice (Hirt et al., 2017). AQP4 receptors activation will result in an influx of water into astrocytes, leading to cytotoxic edema (Figure 4).

## 7 CONCLUSIONS

A reversible lesion in the SCC is recognized on MRI in a wide variety of disease conditions. Thus, it may be better to consider it as a secondary change rather than a characteristic finding associated with mild encephalitis/encephalopathy. If there are reversible lesions in the SCC, the symptoms and prognosis are not necessarily favorable, inasmuch as the neurological symptoms of encephalitis/encephalopathy can vary from absent to severe. These results may suggest that physicians should not diagnose or initiate treatment for encephalitis/encephalopathy merely because of the finding of a lesion in the SCC on MRI. This is because neuroradiological features such as isolated high‐intensity signals on DWI and decreased ADC value of the lesion may be indicative of cytotoxic edema. Findings of previous studies suggest that cytokine‐mediated cytotoxic edema of the SCC through cytokines may be an important pathophysiological mechanism underlying this condition.

## CONFLICT OF INTEREST

The author declares no conflict of interest.

## Data Availability

The data that support the findings of this study were derived from the following resources available in the public domain: Science Direct at https://www.sciencedirect.com/, Wiley Online Library at https://onlinelibrary.wiley.com/, and PubMed at https://www.ncbi.nlm.nih.gov/pubmed. Additional datasets generated and analyzed during the current study are available from the corresponding author on reasonable request.
